# A social network analysis model approach to understand tuberculosis transmission in remote rural Madagascar

**DOI:** 10.1186/s12889-023-16425-w

**Published:** 2023-08-09

**Authors:** Christine Pando, Ashley Hazel, Lai Yu Tsang, Kimmerling Razafindrina, Andry Andriamiadanarivo, Roger Mario Rabetombosoa, Ideal Ambinintsoa, Gouri Sadananda, Peter M. Small, Astrid M. Knoblauch, Niaina Rakotosamimanana, Simon Grandjean Lapierre

**Affiliations:** 1 Stony Brook University, 101 Nicolls Road, Stony Brook, NY 11794-8343 USA; 2 Francis I. Proctor Foundation, University of California, San Francisco, 490 Illinois Street, 2nd Floor, San Francisco, CA 94110 USA; 3 Centre ValBio Research Station, BP 33 Ranomafana, Ifanadiana, Madagascar; 4 Institut Pasteur de Madagascar, 101 Ambohitrakely, Antananarivo Madagascar; 5 Case Western Reserve University, 10900 Euclid Ave, Cleveland, OH 44106 USA; 6 Swiss Tropical and Public Health Institute, Allschwil, Switzerland; 7 University of Basel, Basel, Switzerland; 8 Centre de Recherche du Centre Hospitalier de L, Université de Montréal, 900 Saint-Denis, Montréal, H2X 3H8 Canada; 9 Université de Montréal, 2900 Edouard Montpetit, Montreal, H3T 1J4 Canada

**Keywords:** Tuberculosis, Public Health, Modeling, Social network analysis

## Abstract

**Background:**

Quality surveillance data used to build tuberculosis (TB) transmission models are frequently unavailable and may overlook community intrinsic dynamics that impact TB transmission. Social network analysis (SNA) generates data on hyperlocal social-demographic structures that contribute to disease transmission.

**Methods:**

We collected social contact data in five villages and built SNA-informed village-specific stochastic TB transmission models in remote Madagascar. A name-generator approach was used to elicit individual contact networks. Recruitment included confirmed TB patients, followed by snowball sampling of named contacts. Egocentric network data were aggregated into village-level networks. Network- and individual-level characteristics determining contact formation and structure were identified by fitting an exponential random graph model (ERGM), which formed the basis of the contact structure and model dynamics. Models were calibrated and used to evaluate WHO-recommended interventions and community resiliency to foreign TB introduction.

**Results:**

Inter- and intra-village SNA showed variable degrees of interconnectivity, with transitivity (individual clustering) values of 0.16, 0.29, and 0.43. Active case finding and treatment yielded 67%–79% reduction in active TB disease prevalence and a 75% reduction in TB mortality in all village networks. Following hypothetical TB elimination and without specific interventions, networks A and B showed resilience to both active and latent TB reintroduction, while Network C, the village network with the highest transitivity, lacked resiliency to reintroduction and generated a TB prevalence of 2% and a TB mortality rate of 7.3% after introduction of one new contagious infection post hypothetical elimination.

**Conclusion:**

In remote Madagascar, SNA-informed models suggest that WHO-recommended interventions reduce TB disease (active TB) prevalence and mortality while TB infection (latent TB) burden remains high. Communities’ resiliency to TB introduction decreases as their interconnectivity increases. “Top down” population level TB models would most likely miss this difference between small communities. SNA bridges large-scale population-based and hyper focused community-level TB modeling.

**Supplementary Information:**

The online version contains supplementary material available at 10.1186/s12889-023-16425-w.

## Background

Tuberculosis transmission (TB) models indicate that interventions currently deployed in many countries are insufficient to meet the World Health Organization (WHO) 2035 elimination goals [1–3]. Determining which specific interventions are needed is contingent on understanding context-specific transmission dynamics and drivers of this ongoing pandemic. Accurately describing *M. tuberculosis* epidemiology and understanding its within-population transmission patterns are challenging [4, 5]. Given its airborne route of transmission, index and contacts are difficult to identify with certainty. Also, its incubation period is dependent on host factors including, without being limited to, vaccination status, age, and general immunity state, such that the period between initial infection and progression to active disease is highly variable. This is especially true in high-burden settings with reoccurring exposure to contagious people (TB disease / active TB), which in turn generates high rates of non-contagious but infected patients (TB infection / latent TB / LTBI).

TB transmission models are conventionally constructed as compartmental SEIR (standing for susceptible, exposed (i.e., TB infection), infectious (i.e., TB disease), and recovered) models that include stochastic dynamics to account for time-dependent variance and transition of people between model categories [6–8]. The models are built and calibrated using population-level surveillance data, which can be inaccurate or absent for high-burden rural and remote areas in low-income counties. In such contexts, high-resolution understanding of highly variable local socio-demographic structures, community interconnectivity, and TB infection/disease distribution within those social structures could improve model realism and enhance prediction accuracy of the course of a TB epidemic and impact of control interventions. Social network analysis (SNA) informed by ethnographic data on the nature and structure of contacts between people may offer insights and improve the realism of compartmental models. Common SNA sampling approaches (i.e., snowball sampling from an interviewee’s (“ego”) contacts (“alters”) and cross-linking of places of social aggregation) were previously shown to identify additional TB disease patients and orient TB control workers toward subgroups with higher TB infection rates [9]. Coupled with molecular data, SNA also revealed the importance of ephemeral social contacts and behavioral patterns by showing that transmission occurred between people who could not necessarily name each other [10, 11]. SNA-metrics were not previously used to calibrate and improve an agent-based stochastic TB transmission model.

Madagascar is a high TB burden country where most of the population live in remote rural settings characterized by tight-knit communities and crowded housing. In these settings, healthcare access is limited, disease surveillance systems are underperforming, and only partial TB epidemiological information is available due to significant under diagnosis and under reporting of cases [12]. Also, limited understanding of population dynamics and social interactions, both within and between remote communities, contributes to our inability to understand TB transmission and hence design effective control strategies.

We used SNA to describe five independent and interconnected social networks surrounding patients diagnosed with TB disease in remote Madagascar. We fit network data to exponential random graph models (ERGMs) to generate stochastic, agent-based epidemic simulations of multi-year TB burden within those communities. With this contextualized model, we evaluated the long-term impact of WHO-recommended TB control interventions. We also modelled re-introduction of TB disease following hypothetical TB elimination to estimate resiliency of these isolated communities and excess death due to post-eradication spread.

## Methods

### Overview

We selected 5 TB-affected villages that were representative of Madagascar’s remote context to collect prevalence data on TB infection (i.e., latent TB) and disease (i.e., active TB). We administered individual-level questionnaires to infected patients and their contacts. Contact data were used to construct a social network. Local and global centrality measures were calculated for each village network. Exponential random graph models (ERGM) were fit to identify covariates of tie formation in these networks. We then simulated an SEIR transmission model over the ERGM-derived networks. We calibrated the models on TB infection and TB disease prevalence data. Final models were used to predict long-term TB burden within these communities with and without specific interventions.

### Study villages and participants

**Fig. 1:**
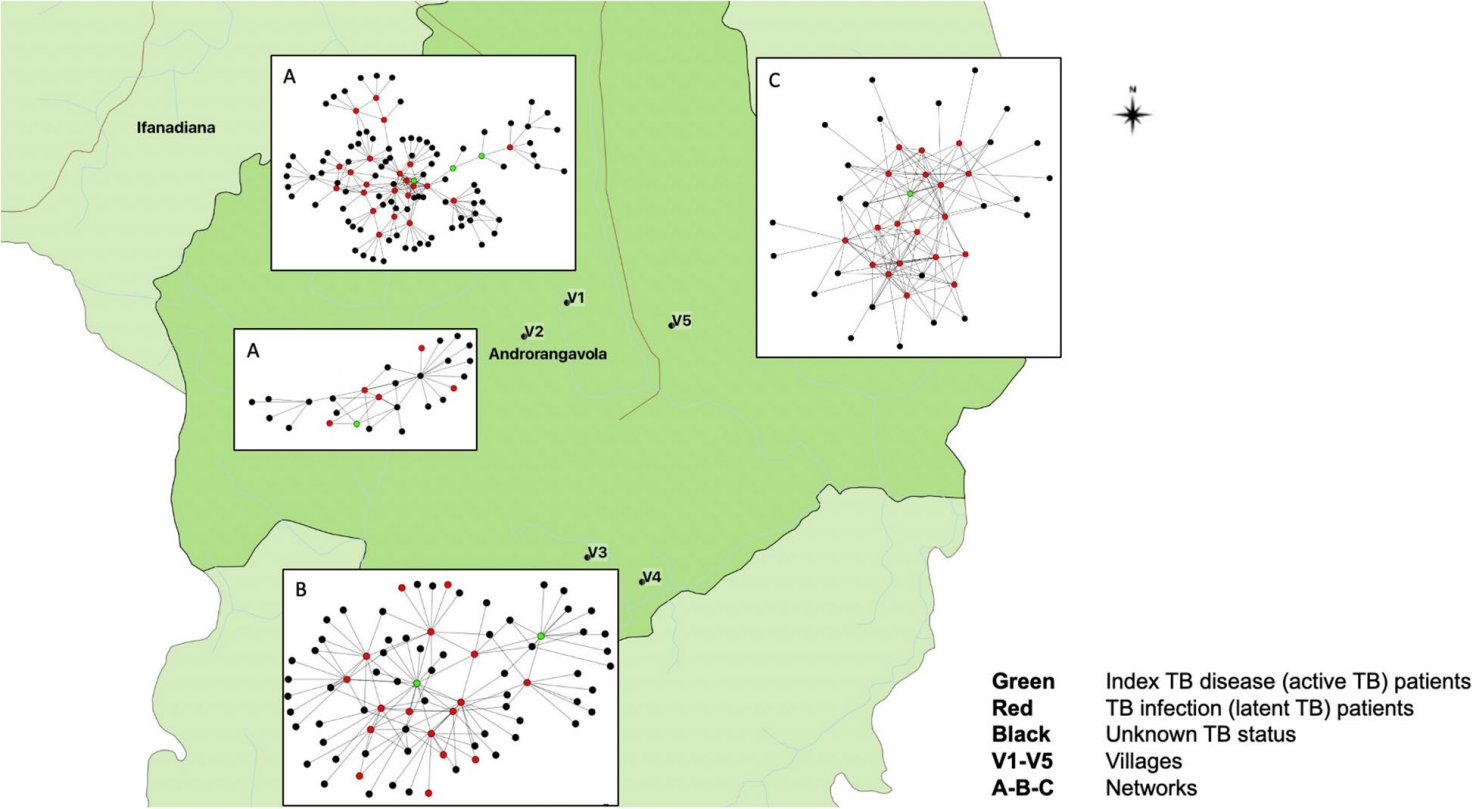
Study area and social network analysis: The Androrangavola commune and social networks created from each village (V1 to V5). Green nodes represent participants with TB disease or those in the “infected” model category, red nodes represent participants with TB infection or those in the “exposed” model category, and black either had negative tests or were untested and are hence confirmed or presumptively in the “susceptible” model category. Villages V1 and V2, represented in Network A, were combined due to geographical proximity and the small size of Village 2. Villages V3 and V4 are represented in Network B and make up a single network because questionnaires revealed they were socially interconnected. Village V5 is represented by Network C

The Androrangavola commune of the Ifanadiana district in southeastern Madagascar (Fig. 1) is an isolated mountainous region that exemplifies Madagascar’s rural demographics and living conditions including young age, large families living in shared households, and high community proximity. Access to healthcare facilities is limited because TB diagnosis occurs at a centralized facility serving approximately 200,000 people. Together with poor diagnostics infrastructure, this contributes to underdiagnosing and underreporting of TB and could partially account for why the reported regional incidence of TB disease is less than one-third of what WHO models predict at country level [12–14]. In Madagascar, fokontanys (hamlets) regroup, on average, 5–6 villages of between 100 and 200 people and represent the lowest administrative and communal structures. To develop representative network-based TB transmission models, we selected five independent villages of the Androrangavola commune in which Madagascar National Tuberculosis Program had previously diagnosed TB patients. All individuals within those communities were eligible to participate in SNA questionnaire data collection and TB prevalence survey. All prevalent TB patients were systematically offered to participate in the study and a house-to-house prevalence survey was also performed to recruit additional participants (see below). Patients under 15 years old or those unable to provide informed consent or participate in the questionnaire due to cognitive impairment were excluded.

### Tuberculosis prevalence survey

All participants recruited during house-to-house visits were tested for TB infection by tuberculin skin test (TST) [15]. All participants presenting with pulmonary-TB-compatible symptoms were tested for TB disease using Xpert MTB/RIF PCR assay (Cepheid, Sunnyvale, CA, USA) on sputum samples [16]. Contacts who did not participate were not tested for TB infection.

### Social network data and sampling

Social networks were constructed from relational datasets comprised of participants with TB disease (“egos”) and their named contacts (“alters”). Egos and alters were connected to each other in the network by edges that represent different types of social contacts (e.g., household contact, regular non-household contact, etc.) and relationships (e.g., family members, close friends, professional contacts, etc.). We first recruited TB disease patients who were labelled as “index cases” (index case here refers to patient initially included in the model and does not infer transmission directionality). Named contacts of index cases were classified as follows: 1) “household contacts,” who sleep in the same house and eat meals together; 2) “close contacts,” who do not share a household but share meals together at least three times a week or spend time together every day; and 3) “other contacts,” who have been named without fulfilling previously mentioned criteria. Using a snowball sampling method, first-degree (i.e., ego’s contact) and second-degree (i.e. contact’s contact) contacts were located and recruited to participate in the same questionnaire and be tested for TB if not previously tested. Some named contacts could be found to complete a questionnaire and be tested for TB. Those who could not be tested for TB were nevertheless included in the network construction. All questionnaires were administered by a study investigator together with a Malagasy healthcare professional and oral translation was performed in real time (Supplementary material 1). Entity resolution, which is the process of identifying when people appear multiple times in the dataset, was completed during the interview by confirming a named contact’s family and location. Because communities were small and close knit, it was feasible to ensure accurate contact information in the dataset.

The ‘igraph’ package for R (version 4.2.2; R Core Team, Vienna Austria) was used to construct social networks and calculate descriptive network statistics [17]. Demographic (e.g., age, sex, profession) and medical (e.g., TB disease, TB infection) data for each participant were incorporated into the network as nodal attributes (i.e., characteristics of network nodes, wherein each node represents a unique person (including egos and alters) in the dataset). Centrality measures and network characteristics for each village, including density (number edges within a network divided by the number of possible edges) and transitivity (clustering phenomenon whereby two alters of an ego also share an edge, which, structurally, forms a closed triad) were calculated.

### Exponential random graph models

The ‘statnet’ package for R was used to generate exponential random graph models (ERGMs) for each network, wherein the coefficients estimated from the ERGMs represent the probability of a tie between two nodes, based on nodal characteristics, while also controlling for network-level properties (e.g., transitivity, edgewise shared partners) [18, 19]. ERGM mathematical notation is defined in [19]; in brief, interpretation is similar to logistic regression, where the dependent variable is binary (tie/no tie), with an important difference: ERGMs allow for the violation of variable independence, which is inherent in relational datasets [19]. Significant nodal and network attributes from the best ERGM for each network served as the base input for TB transmission simulations (Supplementary material 2).

### Network model assumptions and natural history of disease

Progression from infection to disease state was modelled according to fast-slow dynamics as typically used in TB transmission models using previously reported decreasing rates of 38/1000, 3.4/1000, and 0.76/1000 (number of patients progressing to active disease per number of patients with latent infection), respectively, in the first year (rapid progression), two to five years, and the sixth year onward (slow progression) [20, 21]. We assumed that patients with TB disease are not being treated, as is typical for a majority of cases in rural Madagascar, and either recover spontaneously or die. Recovery (218/1000 people per year) and mortality (344/1000 people per year) rates are based on published estimates [22]. Recovered cases have an 80% protection against reinfection [23]. Since HIV prevalence in the study area was previously measured to be zero and since this is the most important risk factor for reinfection, we did not consider reinfections in the model [15]. The birth rate, corresponding to new people entering the model, was estimated as 34.8/1000 per year, based on demographic and health survey data from the Malagasy study region [24]. The death rates of both susceptible and recovered people were estimated to be 4.8/1000 per year. These entry and exit data were calibrated to World Bank Data to achieve an overall population growth of around 2.5% per year [25]. Model parameters, assumptions, and references are presented in Table 1.

**Table 1:** Model parameters

Parameters	Definition	Value	Data source
Primary data			
Act Rate: Number of interactions per week	Average number of transmissible contacts per partnership per week		Averaged from SNA data
Network A		70 per week	SNA Data
Network B		76 per week	SNA Data
Network C		69 per week	SNA Data
Calibrated Data			
Infection Rate (inf)	Probability of infection per transmissible contact between an infected and susceptible individual	Calibrated to 167/1000 per year	Estimated by model calibration
Literature data			
Latency Stage 1: First 12 months (Lf)	First stage of infection: Vector value that is the rate of progression from latent infection to active disease for the first year	38/1000 per year	Menzies et al. [20]
Latency Stage 2: Years 2–5 (Ls1)	Second stage of infection: Vector value that is the rate of progression from latent infection to active disease for years 2—5 after exposure	3.4/1000 per year	Menzies et al. [20]
Latent Slow Progressors: Year 6—15 (Ls2)	Third stage of infection: Vector value that is the rate of progression from latent infection to active disease for years 6—15 after exposure	0.76/1000 per year	Menzies et al. [20]
TB Mortality Rate (TbM)	Rate of people dying from active infection every year	344/1000 per year	Ragonnet et al. [22]
Recovery Rate (TbR)	Rate of people recovering from Active TB every year	218/1000 per year	Ragonnet et al. [22]
Arrival Rate	Birth rate for the region	34.8/1000 per year	Instat-Madagascar 2010 [24]
Death Rate: General Population	Death rate for susceptible, latent infected, and recovered individuals	17/1000 per year	Calibrated from The World Bank [25]

### Model calibration

**Fig. 2:**
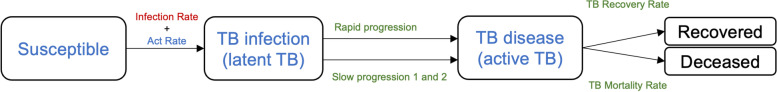
Structure﻿ of tuberculosis SEIR transmission model: Primary data informing the model is shown in blue. This includes act rate, or the number of interactions between egos and alters per week, and prevalence of TB infection (latent TB) and TB disease (active TB). Metrics on the natural evolution of disease extracted from the literature are in green. Infection rate calibrated from community-specific social networks metrics is presented in red. Slow or rapid progression from infection to disease is based on time since initial exposure (see Table 1)

Most TB transmission models are created from a “top down” approach using population data to simulate target patients, communities, populations, or countries. [7] Fictive individuals are randomly generated and assigned relevant epidemiological characteristics that influence contact rates and transitions to different infection states. Our models were constructed using a “bottom up” approach by fitting individual attributes and relational structures from SNA on primary TB prevalence data. We built a stochastic network-based model with four infection stages (i.e., SEIR) that reflect the natural history of TB: 1) “susceptible,” no prior history of TB disease, negative TST, and either no active symptoms or a negative Xpert test; 2) “exposed,” TB infection defined by a positive TST and negative Xpert test; 3) “infected,” TB disease defined by positive Xpert test; and 4) “recovered,” a documented history of bacteriologically confirmed TB disease followed by either treatment or recovery. Individuals move across categories according to rate parameters in Table 1. The probability of “susceptible” individuals becoming “exposed” (TB infection) is a function of infection probability and contact rate. The contact rate is dynamic and varies according to tie likelihood between any two nodes with characteristics in the best-fit ERGM for each village. Infection rate was estimated by calibrating the model to achieve the estimated TB infection (latent TB) burden in the same villages [14]. The highest risk of progression from “exposed” (TB infection) to “infected” (TB disease) occurs in the first year and decreases over time. Individuals either remain in this category until the end of simulations, move to the “infected” (TB disease) category, or exit the model when they die of non-TB-related causes [20]. Simulations were run in time steps of a week because contact classifications in our field surveys were based on weekly interactions. Primary model outcomes included prevalence of TB infection, TB disease, number of recovered individuals, and TB mortality. Models were calibrated independently for each village network. Calibration simulations were run for 40 years with the goal of achieving an equilibrium state reflecting TB infection and TB disease prevalence as measured in the prevalence survey. The ‘EpiModel’ package for R was used to simulate transmission dynamics over a stochastic network derived from our ERGM simulations (Fig. 2). [26]. EpiModel includes all disease structures (e.g., SI, SIS, SIR), with user-defined inputs for SEIR-structured transmission, such as fast-slow stochastic transition likelihoods from latent to infected states. Underlying algorithms for network and transmission simulation are available in [26] and we included our model code as supplementary materials 4.

Achieving a steady state was challenging, particularly because the prevalence of exposed patients consistently decreased despite high infected rates. Our estimate from field data of 78% TB infection (latent TB) prevalence could be an overestimate due to our sampling strategy in which snowballing from TB disease patients might lead to oversampling of participants with TB infection in the dataset. We decreased the prevalence of latent TB infection at t_0_ to 68% to account for this, which yielded a difference between model-generated prevalence at steady state and observed prevalence of around 10 to 15 fewer individuals with TB infection than expected when the infection rate was set to 167/1000 people per year (Supplementary material 4). This calibration led to the closest estimates to our measured prevalence.

### Model scenarios

Calibrated models were then used to simulate specific scenarios of public health and TB control interest (Table 2). Scenario 1 (baseline model) simulates the natural history of TB in all villages with zero interventions over the course of 15 years with 2035 as the endpoint i.e., the baseline model. Scenario 2 (intervention model) assesses the feasibility of achieving the WHO 2035 elimination goals with currently recommended strategies. This is an intervention model representing active case finding and treatment within villages for a 15 years period. In this scenario, we assumed that 98% of all TB disease cases were no longer infectious after six months. Scenario 3 (resiliency model) assesses how resilient communities would be to re-introduction of TB after hypothetical elimination. In this scenario, we assumed the population had previously been treated to the point of being TB naïve and initial parameters were set so that 98% of the population was susceptible and no one had active TB disease. This scenario assessed how TB can spread through communities under two different scenarios: introduction of a single case versus introduction of three cases of TB disease. Scenario 4 (long-term model) represents the natural history of TB with no interventions over 65 years to show the natural course of TB throughout the villages and assesses the ability of the model to predict long-term TB dynamics and estimate excess mortality due to untreated TB disease.

**Table 2:** Model scenarios

Scenario	Purpose	Time	Baseline Value	Scenario specific parameters
Baseline model	Simulate natural evolution of TB	15 years	Baseline parameters	None
Intervention model	Simulate Active Case Finding and Treatment Assess achievability of End TB goals	15 years	*Recovery rate* 218/1000 per year	*Recovery Rate* 980/1000 per 6 months
Resiliency model	Assess resiliency of community to reintroduction of active disease	15 years	*SEIR distribution* 30% of population susceptible	*SEIR distribution* 98% of population susceptible
Long-term model	Assess the natural history of TB over a Malagasy life expectancy under baseline conditions	65 years	Baseline parameters	None

## Results

### Study population, setting, and TB prevalence

Participant sociodemographic characteristics and TB disease status are presented in Table 3. Mean participant age was 32.2 years old and the male:female ratio varied by village. A large majority of participants were subsistence farmers. All but two participants lived in one-room traditional mud houses where household members socialized, cooked, ate, and slept. Household size ranged from 3 to 10 people. TB disease (active TB) was diagnosed among seven patients across the five included villages. Prevalence of TB infection (latent TB) was 78%.

**Table 3:** Participants and networks characteristics

	Network A	Network B	Network C
	Village 1 & 2	Village 3 & 4	Village 5
	(*n* = 107)	(*n* = 118)	(*n* = 45)
Participants characteristics			
Demographics			
Mean age in years (SD)	33 ± 16	32.6 ± 15.3	31.1 ± 13.7
Male	54 (50.5%)	67 (56.8%)	17 (37.8%)
Female	53 (49.6%)	51 (43.2%)	28 (62.2%)
Occupation			
Farmer	92 (86.0%)	100 (84.7%)	38 (84.4%)
Student	6 (5.6%)	6 (5.1%)	5 (11.1%)
Community health worker	1 (0.9%)	1 (0.8%)	0 (0.0%)
Teacher	4 (3.7%)	4 (3.4%)	0 (0.0%)
Blacksmith	1 (0.9%)	0 (0.0%)	0 (0.0%)
Business owner	1 (0.9%)	3 (2.5%)	0 (0.0%)
Local midwife	1 (0.9%)	0 (0.0%)	0 (0.0%)
Cattle keeper	1 (0.9%)	0 (0.0%)	1 (2.2%)
Elder	0 (0.0%)	1 (0.8%)	0 (0.0%)
King of the village	0 (0.0%)	1 (0.8%)	1 (2.2%)
President of the fokotany	0 (0.0%)	1 (0.8%)	0 (0.0%)
Tuberculosis status			
Active cases	3	3	1
Latent TB cases	23	35	19
TST tested	30	48	20
TST positivity rate	76.6	72.9	95.0
Network characteristics			
Nodes	107	118	45
Edges	165	197	151
Density	0.029	0.029	0.15
Transitivity	0.16	0.29	0.43
Mean Degree	6.2	6.7	13.4

*SD* Standard deviation, *TB* tuberculosis, *TST* tuberculin skin test; Nodes: individuals, Edges: connections between individuals, Density: the number of actual ties in a network divided by the number of potential ties, Transitivity: the clustering phenomenon whereby two alters of an ego also share a tie, which, structurally, forms a closed triad

### Social network analysis

The 83 egos generated 187 alters for a total of 270 network nodes. Villages V1 and V2, represented in Network A, were combined due to geographical proximity and the small size of Village 2. Villages V3 and V4 are represented in Network B and make up a single network because questionnaires revealed they were socially interconnected. Village V5 is represented by Network C (Fig. 1, Supplementary material 3). All three networks showed high levels of interconnectivity between participants, with network densities of 0.029, 0.029, and 0.15, respectively, and transitivity (i.e., triad closure) values of 0.16, 0.29, and 0.43, respectively (Table 3).

### Impact of WHO-recommended interventions

**Fig. 3:**
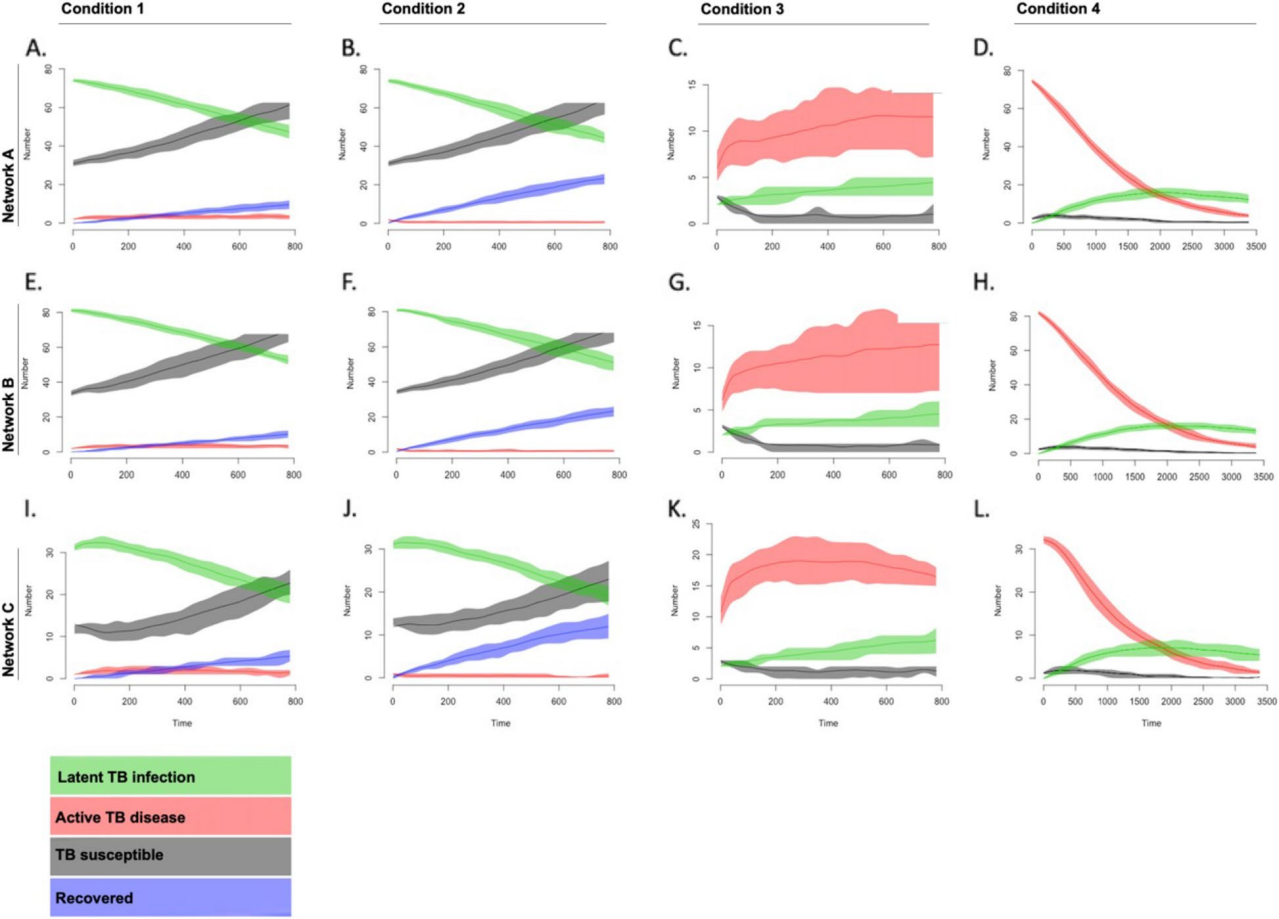
Tuberculosis evolution modeling at community level Impact of model conditions on social networks

Active case finding and treatment (Scenario 2) over 15 years led to significant decrease in TB disease prevalence compared with the baseline model (Table 4). At the end of the simulation, Network A had 0 disease (active TB) cases and 44 infection (latent TB) cases. Network B had 0 disease cases and 51 infection cases. Network C had 0 disease cases and 20 infection cases. There was a 79%, 75%, and 67% reduction in TB disease prevalence in Networks A, B, and C, respectively. Meanwhile, latent TB infection prevalence only slightly declined by 6.1%, 2.3%, and 4.4%, respectively, in Networks A, B, and C. TB mortality decreased in all three networks with active case finding and treatment, with Network B experiencing the largest reduction at 78%, Network C at 75%, and Network A at 73% (Table 4 and Fig. 3).

**Table 4:** Tuberculosis transmission models and interventions predictions

	Active TB proportion	Recovered proportion	TB Mortality proportion	LTBI proportion
Network A				
Starting prevalence	1.9	-	-	67.0
Original	2.9 ± 1.4	7.9 ± 2.4	14.0 ± 3.6	39 ± 4.0
Intervention	0.6 ± 0.6	17.7 ± 3.5	3.4 ± 1.8	33.5 ± 3.3
65 Years	0.2 ± 0.3	4.7 ± 1.4	14.9 ± 2.0	1.5 ± 0.7
1 reintroduction	0.2 ± 0.5	1.9 ± 0.9	1.3 ± 1.2	3.5 ± 2.7
3 reintroduction	0.7 ± 0.8	3.3 ± 1.4	4.1 ± 2.1	8.7 ± 4.5
Network B				
Starting prevalence	1.7	-	-	68.0
Original	2.4 ± 1.6	7.6 ± 2.5	13.7 ± 4.1	38.7 ± 3.2
Intervention	0.6 ± 0.7	16.2 ± 3.4	2.8 ± 1.3	35.3 ± 3.4
65 Years	0.1 ± 0.2	4.6 ± 1.0	14.5 ± 1.8	1.4 ± 0.6
1 reintroduction	0.4 ± 0.6	2.0 ± 1.2	1.4 ± 1.7	4.6 ± 5.1
3 reintroduction	0.6 ± 0.7	3.1 ± 1.3	3.5 ± 2.3	8.7 ± 4.4
Network C				
Starting prevalence	2.2	-	-	67.0
Original	3.0 ± 2.9	10.6 ± 4.4	19.7 ± 7.6	40.9 ± 6.9
Intervention	0.9 ± 1.2	22.2 ± 6.9	4.6 ± 3.4	35.3 ± 6.3
65 years	0.2 ± 0.4	5.2 ± 2.1	17.2 ± 3.2	1.3 ± 1.1
1 reintroduction	2.1 ± 2.4	7.5 ± 3.4	7.3 ± 5.2	27.2 ± 11.9
3 reintroduction	2.7 ± 2.4	12.8 ± 5.2	15.6 ± 7.0	33.7 ± 7.0

*LTBI* latent tuberculosis infection, *TB* tuberculosis

### Community resilience to TB disease

TB disease prevalence in Networks A and B remained under 1% for all simulations, and the highest prevalence of latent TB infection reached was 8.7%. Mortality in those networks was around 1.3% after a single case introduction. Mortality increased to 4.1% and 3.5% in Networks A and B, respectively following three new case introductions. In Network C, TB disease prevalence was > 2% in both scenarios. Mortality was also higher than in the other networks at 7.3% and 15.6% following one and three new case introductions, respectively. Latent TB infection prevalence increased to 27.2% and 33.7%, returning to levels estimated in models in which no intervention was undertaken (Fig. 3).

### Long-term evolution of TB disease

The generation models were run for 65 years with no interventions to simulate long-term TB dynamics (scenario 4). Initial parameters for each network matched baseline prevalence from our field-informed estimates with no new introductions of TB from outside sources. In all three networks, TB disease prevalence reduced to 0.2% or lower and latent TB infection decreased to 1.5% or lower. Mortality was high in all three networks, 14.9%, 14.5%, and 17.2% in Networks A, B, and C, respectively (Fig. 3).

## Discussion

SNA methods were previously used in contact tracing campaigns or to descriptively understand TB transmission in specific settings. [7–11, 27, 28] We believe this is the first study that leverages SNA-metrics to calibrate and improve an agent-based stochastic TB transmission model. Our innovative approach allowed us to confirm and measure discrepancies in TB transmission dynamics between networks and highlights the added value of the SNA approach to increase the accuracy of prediction models and the resolution of outcomes at community level. Although the study communities have many similarities, such as geography, demographics, and livelihoods, our SNA approach highlighted important social variations (e.g. density and transitivity) that drive differences in disease transmission and burden.

Although our models showed a considerable reduction in the burden of TB disease (active TB) and TB mortality rates following active case finding and treatment, the WHO’s End TB targets were not reached on a 15-year projection [1]. The prevalence of TB infection (latent TB) only slightly decreased over 15 years of intervention confirming the persistence of TB reservoirs after systematic treatment of active cases. Although it is not currently part of Madagascar’s control protocol, models suggest a potential role for TB preventive therapy (TPT) as has been previously suggested in other settings [29, 30]. Although elimination is not yet achieved, we used the models to assess community “resilience,” or how easily TB disease could re-establish in TB-naïve communities. This is of particular importance given the vertical nature of TB-specific programs, which often consists of temporally and geographically focused TB elimination campaigns for communities that are served by poorly developed universal health systems. We tested this in our 15-year resiliency simulations. In networks A and B, when three new active cases of TB were introduced at T_0_, the burden of TB disease remained below 200/100,000 with a mortality rate of 4,100/100,000. However, latent TB infection remained at 8,700/100,000, which is concerning, because although this is a decrease from current estimates, it nevertheless indicates a remnant disease reservoir was not eliminated. Network C showed lower resilience against active TB disease, reaching 2,700/100,000. TB infection prevalence and mortality were also higher than in Networks A or B. Network C exhibits higher structural centrality as shown in density and transitivity values, indicating that people in that community, overall, have larger social networks. Density refers to the number of ties within a network divided by the number of potential ties. Transitivity is the clustering phenomenon whereby two alters of an ego also share a tie, which, structurally, forms a closed triad. This is also reflected in the higher mean degree, which is the mean number of connections each person in the network has. Therefore, there are more opportunities in Network C to be exposed to TB compared with the other networks, indicating that intensity of social contacts might foster greater exposure to TB. Such inter-community differences would not be captured by conventional transmission models.

Our study had several limitations. Sample size was limited, and inclusions were opportunistic, meaning that we prioritized contacts according to willingness to participate and potential TB symptoms. Many contacts lived in barely accessible field houses. Individuals under 15 years old were not included and could have equally contributed to disease transmission. This might have biased individuals’ representation in our models. Estimating time and frequency of contacts was sometimes challenging, especially in the context of close and frequent contacts. This may have influenced the density metrics of community networks. We did not collect sputum from asymptomatic participants, so we may have underestimated TB disease prevalence. In parameterizing our models, we did not incorporate the possibility for reinfection. This clinical scenario is mostly encountered among immunocompromised patients, such as those with HIV or diabetes mellitus, and data from the study area showed 0% HIV prevalence and type-II diabetes mellitus prevalence was unknown [15].

Latent TB infection in scenario 4 (i.e., 65 years projection) decreased to very low prevalences and we were not able to simulate the prevalences we estimated from our field study. In the models, our starting prevalences, represented by the initial conditions, were meant to reflect the community baselines given that there has been no TPT administration or other treatment strategies to diagnose and treat latent TB infection. Without any LTBI treatment, we would expect the TB burden to remain relatively steady across a generation. However, we could not replicate this in our models and, rather, LTBI declined over time and never reached a steady state, even after a 65-year simulation. This could be because we did not take into consideration individual mobility and outside sources of transmission. In the survey data, 88% of the population reported traveling to the commune center for market day at least once a week. Some participants also reported traveling to outside villages at least once a year. These frequent or socially intense mobilities could be important for TB transmission and importation into village communities and could be particularly important for maintaining high levels of LTBI that we could not capture in our models without incorporating spatial dynamics.

## Conclusion

SNA highlights community-specific differences in social structures. SNA-informed stochastic models enable higher resolution and community-specific modeling of TB control interventions. More comprehensive and longitudinal characterization of setting-specific social dynamics and better data on regional mobility and contact patterns when traveling (e.g., frequency and intensity of close contacts) are needed. These insights would improve models further and would highlight the expected community-specific impact of recommended approaches to TB elimination.

### Supplementary Information

**Additional file 1.** 

**Additional file 2.** 

**Additional file 3.** 

**Additional file 4.** 

**Additional file 5.** 

## Data Availability

All primary data is accessible in the referenced sources in Table 1 or upon request directly to the corresponding author.

## Abbreviations

CERBM: Comité d’éthique à la Recherche Biomédicale
ERGM: Exponential random graph model
FRQS: Fonds de Recherche Québec Santé
IRB: Institutional Review Board
LTBI: Latent Tuberculosis Infection
PCR: Polymerase Chain Reaction
SEIR: Susceptible exposed infected recovered
SI: Susceptible infected
SIR: Susceptible infected recovered
SIS: Susceptible infected susceptible
SNA: Social Network Analysis
Tuberculosis: TB
TPT: Tuberculosis preventive therapy
TST: Tuberculin skin test
WHO: World Health Organization
