# Supplementary material for: A social network analysis model approach to understand tuberculosis transmission in remote rural Madagascar
Source: BMC Public Health. 2023 Aug 9;23:1511. doi: 10.1186/s12889-023-16425-w (PMC10410943; doi:10.1186/s12889-023-16425-w)
Supplement: Supplementary file 1 — Additional file 1. [file 12889_2023_16425_MOESM1_ESM.docx]

**SOCIAL NETWORK ANALYSIS IN REMOTE COMMUNITIES OF MADAGASCAR**

**Title of Study:** Connecting the DOTS: A Social Network Analysis to TB Transmission in Remote Communities

**IRB Number: __________________________________**

**PI: _ _______________**

**Participant ID number ____________________**

**Date of Visit ______ / _______ / ___________**

**Location of Visit: _______________________**

**Initials: ______________________________**

**INSTRUCTIONS**

**Please follow all instructions and complete study activities in the order included in the packet.**

**All questions and answers will be written with BLACK pen**

**Please check that all questions are completed and that the visit information is filled out at the top of each page.**

**ELIGIBILITY AND CONSENT**

**READ: Thank you for considering participating in our research. In order to move forward, I need to ask you a few medical history questions to determine if you are eligible for participating.**

**The following must be circled YES to proceed:**

1) Participant is not refusing to sign the informed consent Y N

*If the participant is a child (<18 years), the guardian must provide consent and the child assent. Questions will be answered by the guardian for the child*

2) Participant is from Madagascar Y N

**The patient is eligible. The following criteria must be circled YES to proceed:**

1. Consent Form is verified IRB approved and current Y N Date: ___/___/___

2. Subject had consent form read to them Y N

3. Subject understands the purpose, risk and benefits of study participation Y N

4. Consent was appropriately signed prior to any study procedures being performed Y N

5. Subject was provided a copy of the signed informed consent Y N

6. Subject was given contact information to call with any questions regarding the study Y N

Consent obtained by:

_______________________ ____________________ ____/____/____

(Print name) (Signature) (Date)


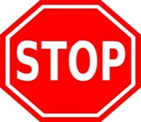


****DO NOT PROCEED TO QUESTIONNAIRES UNLESS THE CONSENT PROCESS HAS BEEN APPROPRIATELY CONDUCTED AND THE CONSENT FORM SIGNED****

**I - Demographics**

D1) Commune: ________________ D2) Fokontany: ________________

D2) Village: ________________

D3) Age (in years): ________________

D4) Date of birth: (DD/MM/YYYY) ________________

D5) Gender: M / F

D6) Family: Number of people in the household: ____

D7) Confirmed TB patient: Y / N

**II - Social Behaviors**

Household

S1) Describe where and how people sleep in your house: ________________________________________________________________________

S2) Describe how meals are shared: _____________________________________________________________________________

S3) Have any of the people living with you traveled outside the village in the last month? ______________________________________________________________________________

S4) Where did they travel to? _____________________________________________________________________________

Interpersonal Relationships

S5) Describe how you greet friends and family when you see them? ________________________________________________________________________

Mobility

S6) How many houses do you have? _____

S7) Do you travel outside the village? Y / N

S8) Do you spend more time in the field or in the village? F / V

S9) How often do you travel outside the village? ________________________________________________________________________

S10) When were your last two trips? ________________________________________________________________________

S11) How many days was your last stay outside the village? ________________________________________________________________________

S13) Describe where you sleep when you are outside the village: ________________________________________________________________________

S14) How many other people sleep in the same room as you: ________________________________________________________________________

**III- Individual Contact Generating Questions**

*For each answer collect: Name, Surname, Alternative Names, Age, Job, Relationship*

*Limit Response to top 5 people*

Household

C1) Who sleeps in the same room as you at least 3 nights a week?

________________________________________________________________________

C2) Who do you eat meals with?

________________________________________________________________________

Interpersonal Relationships

C3) Who do you share intimacy with?

________________________________________________________________________

C4) Who do you spend time inside with more that 3 days a week?

________________________________________________________________________

Mobility

C5) Who did you specifically travel to meet outside the village in the last month?

________________________________________________________________________

C6) Who do you travel outside the village with?

________________________________________________________________________

**IV- Specific Social Aggregation Questions**

*For each location collect: specific location, surrounding landmarks, GPS coordinates*

*Limit each response to the top 3 places*

SA1) Where do you live? ______________________________________________________________________________

SA2) Where do you eat meals at least 3 times a week? ______________________________________________________________________________

SA3) Where do you interact with named contacts inside the village?

______________________________________________________________________________

SA4) How many days a week do you visit the locations named above?

______________________________________________________________________________

SA5) Where are the 3 places you most interact with people outside the village?

______________________________________________________________________________

SA6) Where do you stay when you are outside the village? (Specific location w/ landmark)

______________________________________________________________________________
