# Supplementary material for: A social network analysis model approach to understand tuberculosis transmission in remote rural Madagascar
Source: BMC Public Health. 2023 Aug 9;23:1511. doi: 10.1186/s12889-023-16425-w (PMC10410943; doi:10.1186/s12889-023-16425-w)
Supplement: Supplementary file 2 — Additional file 2. [file 12889_2023_16425_MOESM2_ESM.docx]

|  | | | | |
| --- | --- | --- | --- | --- |
| **Supplemental Table 2: Network and node attributes of interactions in exponential random graph models** | | | | |
|  | |  | | |
|  | | Network A | Network B | Network C |
|  | | Coef (SE) | Coef (SE) | Coef (SE) |
|  | | | | |
| **Network attributes** | | | | |
| Edges^1^ | -2.2^***^ (0.29) | | -4.7^***^ (0.36) | -2.9^***^ (1.04) |
| GWESP^2^ | 0.5^***^ (0.13) | | 0.9^***^ (0.62) | 3.0^***^ (0.98) |
| Degree (1)^3^ | 2.2^***^ (0.36) | | 2.8^***^ (0.44) | 3.9^***^ (1.0) |
| GWDEG^4^ | -- | | 2.4^***^ (0.68) | -- |
|  |  | |  |  |
| **Node attributes** | | | | |
| **Ego-alter nodes^†^** |  | |  |  |
| Ego=alter | -0.9 (0.15) | | -1.4^***^ (0.2) | -2.2^***^(0.4) |
| **Role** |  | |  |  |
| Uniform homophily (UH)^5^ | -- | | -0.9^***^ (0.2) | -1.0^*^ (0.4) |
| **Gender** |  | |  |  |
| UH | 0.4^*^ (0.19) | | 0.3^*^ (0.1) | -- |
| **Village** |  | |  |  |
| UH | -- | | 2.1^***^ (0.3) | -- |
| AIC | 849 | | 1308 | 617.2 |
| BIC | 880 | | 1362 | 641.7 |
|  |  | |  |  |
| ^*^p<0.05, ^**^p<0.01, ^***^p<0·001  ^†^This is a control variable to account for named contacts having very low degree because, due to not being interviewed, their contacts are largely missing.  ^1^Edges: Number of edges in network. Functions like regression intercept.  ^2^GWESP: geometrically weighted edgewise shared partners.  ^3^Degree(1): number of network nodes with degree=1.  ^4^GWDEG: geometrically weighted degree distribution.  ^5^Uniform homophily: an average rate of tie formation between similar nodes. | | | | |
|  | | | | |
