# Supplementary material for: A social network analysis model approach to understand tuberculosis transmission in remote rural Madagascar
Source: BMC Public Health. 2023 Aug 9;23:1511. doi: 10.1186/s12889-023-16425-w (PMC10410943; doi:10.1186/s12889-023-16425-w)
Supplement: Supplementary file 4 — Additional file 4. [file 12889_2023_16425_MOESM4_ESM.docx]

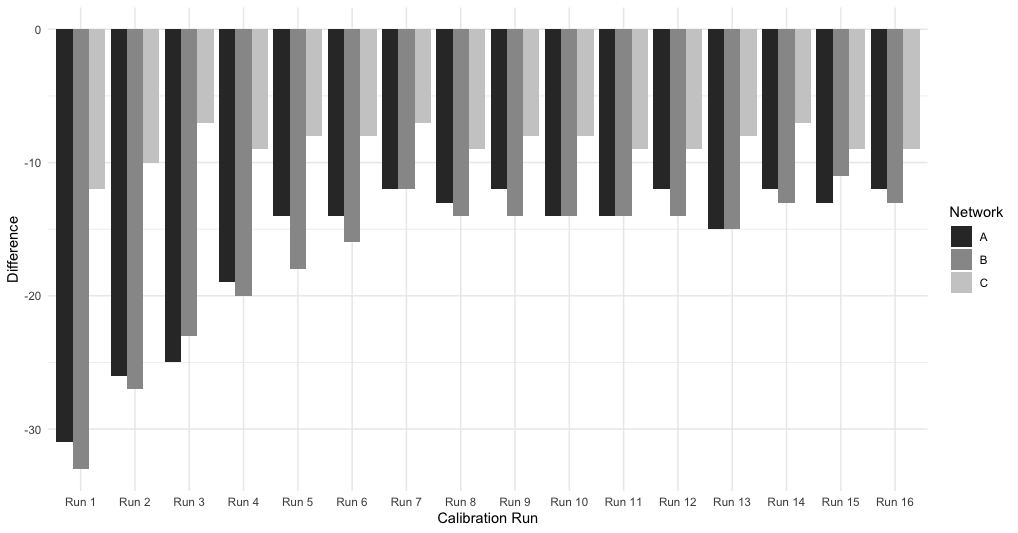


Difference between simulated and observed TB infection (latent TB) prevalence in each run of the calibration models.
