# Supplementary material for: A social network analysis model approach to understand tuberculosis transmission in remote rural Madagascar
Source: BMC Public Health. 2023 Aug 9;23:1511. doi: 10.1186/s12889-023-16425-w (PMC10410943; doi:10.1186/s12889-023-16425-w)
Supplement: Supplementary file 5 — Additional file 5. [file 12889_2023_16425_MOESM5_ESM.docx]

### Network A visualization by TB infection

Legend: Green – Active Pulmonary TB, Red – Latent TB Infection, Black – Unknown Infection Status

### Network B visualization by TB infection

Legend: Green – Active Pulmonary TB, Red – Latent TB Infection, Black – Unknown Infection Status

### Network C visualization by TB infection

Legend: Green – Active Pulmonary TB, Red – Latent TB Infection, Black – Unknown Infection
